# Botulinum neurotoxin serotype A inhibited ocular angiogenesis through modulating glial activation via SOCS3

**DOI:** 10.1007/s10456-024-09935-7

**Published:** 2024-06-26

**Authors:** Austin T. Gregg, Tianxi Wang, Manon Szczepan, Enton Lam, Hitomi Yagi, Katherine Neilsen, Xingyan Wang, Lois E. H. Smith, Ye Sun

**Affiliations:** Department of Ophthalmology, Boston Children’s Hospital, Harvard Medical School, Boston, MA 02115 USA

**Keywords:** Botulinum neurotoxin serotype A, Choroidal neovascularization, Age-related macular degeneration, Glial activation, Müller activation, Microglial activation, SOCS3

## Abstract

**Background:**

Pathological angiogenesis causes significant vision loss in neovascular age-related macular degeneration and other retinopathies with neovascularization (NV). Neuronal/glial-vascular interactions influence the release of angiogenic and neurotrophic factors. We hypothesized that botulinum neurotoxin serotype A (BoNT/A) modulates pathological endothelial cell proliferation through glial cell activation and growth factor release.

**Methods:**

A laser-induced choroidal NV (CNV) was employed to investigate the anti-angiogenic effects of BoNT/A. Fundus fluorescence angiography, immunohistochemistry, and real-time PCR were used to assess BoNT/A efficacy in inhibiting CNV and the molecular mechanisms underlying this inhibition. Neuronal and glial suppressor of cytokine signaling 3 (SOCS3) deficient mice were used to investigate the molecular mechanisms of BoNT/A in inhibiting CNV via SOCS3.

**Findings:**

In laser-induced CNV mice with intravitreal BoNT/A treatment, CNV lesions decreased > 30%; vascular leakage and retinal glial activation were suppressed; and *Socs3* mRNA expression was induced while vascular endothelial growth factor A (*Vegfa*) mRNA expression was suppressed. The protective effects of BoNT/A on CNV development were diminished in mice lacking neuronal/glial SOCS3.

**Conclusion:**

BoNT/A suppressed laser-induced CNV and glial cell activation, in part through SOCS3 induction in neuronal/glial cells. BoNT/A treatment led to a decrease of pro-angiogenic factors, including VEGFA, highlighting the potential of BoNT/A as a therapeutic intervention for pathological angiogenesis in retinopathies.

**Supplementary Information:**

The online version contains supplementary material available at 10.1007/s10456-024-09935-7.

## Introduction

Age-related macular degeneration (AMD) is a major cause of central vision loss among the elderly [1]. In the neovascular (NV) form of AMD, vision loss from choroidal neovascularization (CNV) is rapid and severe [2]. CNV affects ~ 10% of AMD patients, but accounts for up to 90% of vision loss associated with AMD [3]. AMD therapy is centered on intraocular injections of anti-vascular endothelial growth factor (anti-VEGF) therapeutics, which significantly improve and delay the course of NV (wet) AMD. While these treatments are the standard of care, repeated intraocular injections over prolonged periods are required, raising concerns about long-term outcomes [4] including decreased blood flow, and possible aggravation of geographic atrophy [5]. Exploring novel therapeutic approaches is important to address current treatment limitations.

Early rod photoreceptor death is observed in both geographic atrophy (dry) AMD and NV (wet) AMD. Photoreceptor neuron death triggers Müller glia activation [6, 7] but the underlying mechanism is largely unknown. Glial activation (gliosis) is a ubiquitous response to almost every form of retinal injury and disease and is seen in NV AMD, diabetic retinopathy, and retinopathy of prematurity [8]. Activated glial cells release proangiogenic factors which lead to NV and are a major source of VEGF in ischemic retinas [9, 10]. Therefore, the neuronal/glial-vascular communications causing the release of angiogenic and neurotrophic factors play very important roles in controlling CNV.

Botulinum neurotoxin type A (BoNT/A) has been used clinically to treat more than 20 medical conditions, including dystonia, spasticity, pain, migraines, overactive bladder, osteoarthritis, and wound healing [11]. The toxicity and effective doses of BoNT/A have been well studied. BoNT/A is a potent therapeutic agent for diverse motor and autonomic neurologic disorders. The therapeutic efficacy in many of these conditions lies in the cleavage of soluble NSF-attachment protein receptor (SNARE) protein complex which is crucial for vesicular neuroexocytosis and primarily targets peripheral neuromuscular junctions. BoNTs inhibit acetylcholine (ACh) release from cholinergic neurons, thereby disrupting neuronal synaptic transmission. Prior studies have shown BoNT efficacy in chronic neuropathic pain is through inhibition of the release of neurotransmitters other than ACh, such as glutamate, substance-P, and calcitonin gene-related peptide [12]. In vivo studies using mouse models have shown BoNT/A to have mixed effects on angiogenesis in non-ocular tissues. For example, Tang et al. [13] demonstrated improved long-term weight and volume retention of allogeneic adipose tissue transplantation after BoNT/A injections. In this setting, BoNT/A was found to enhance angiogenesis via vasodilation and endothelial proliferation with increased expression of VEGF [13]. Similarly, Koo and colleagues found that BoNT/A administration enhanced the capacity of endothelial cell tube formation and sprouting, thereby promoting vessel formation in mouse models investigating endometrial angiogenesis [14]. Conversely, Zhou et al. found that BoNT/A injections reduce angiogenesis via inhibition of VEGF expression and thereby limiting hypertrophic scar formation in rabbit ear scar models [15]. However, the role of BoNT/A in retinal angiogenesis remains largely unexplored. Further investigation is necessary to understand the specific mechanisms underlying the tissue-dependent angiogenic effects of BoNT/A.

BoNT/A binds to specific receptors on the surface of the presynaptic terminals of neurons and glial cells via its C-terminal receptor binding domain, and initiates an endocytic process, followed by the cleavage of synaptosomal nerve-associated protein 25 (SNAP25) needed for vesicle docking on the nerve terminal to block neurotransmitter release [16]. SNAP25 is involved in photoreceptor differentiation and regeneration in salamanders [17] and is highly expressed in photoreceptor neurons [18, 19]. SNAP25 heterozygous mutants with lower expression shows increased rod photoreceptor differentiation in early retinal development. In salamanders SNAP25 is expressed in normal photoreceptors but not in regenerating photoreceptors [17].

Retinal glial cells are known to undergo morphological and functional changes (glial activation and dysfunction) in disease conditions. Activated glial cells express proangiogenic factors in response to pathogenic stimuli in retinal vascular diseases, including NV AMD [8]. There are three main types of glial cells in the mammalian retina: Müller glia, astrocytes, and microglia [20]. These glial cells provide both structural and functional support to neurons and retinal vessels [20]. Although the exact mechanism is unknown, evidence indicates that BoNT/A can modulate glial cells. Feng et al. observed that BoNT/A effectively impedes spinal glial cell activation and subsequent release of pro-inflammatory factors [21]. BoNT/A interacts with Toll-like receptor 2 (TLR2) in microglia to exert anti-inflammatory effects [22, 23] but appears to have only a slight effect on astrocytes *in vitro.* However, the full activation of TLR2 in astrocytes appears to require the presence of functional TLR4 in microglia [22], suggesting synergistic roles of multiple cell types in vivo [22, 24]. Activated retinal Müller glia express TLR2 and contribute directly to retinal innate response via production of inflammatory mediators [25]. These findings indicate that BoNT/A has a therapeutic potential in pathological conditions caused by glial cell activation.

Here, we used mouse with laser-induced CNV as pathological NV model to investigate BoNT/A’s therapeutic anti-angiogenic effects. NV formation after intravitreal injections of BoNT/A were assessed with immunohistochemistry and fundus fluorescence angiography (FFA). We found that BoNT/A significantly decreased pathological CNV, blood leakage from CNV, and retinal glial activation in the laser-induced CNV mouse model. The protective effect of BoNT/A was absent in suppressor of cytokine signaling 3 (SOCS3) neuronal/glial deficient mice. SOCS3, a member of the SOCS family proteins, has been found to play a pivotal role in regulating the inflammatory response within ocular diseases. Its impact on pathological ocular angiogenesis has been extensively studied [26, 10, 27–30]. Prior research has investigated the anti-angiogenic effects of neuronal and glial SOCS3 in OIR mice, primarily by regulating VEGFA [10]. Additionally, myeloid SOCS3 has been shown to regulate laser-induced CNV by modulating the accumulation of macrophage and microglia [30] as well as in the regulation of OIR via SPP1 [27]. Our findings suggest that BoNT/A may induce SOCS3 in neuronal and glial cells to suppress glial cell activation leading to a decrease of VEGFA and other inflammatory factors.

## Methods

### Animals

All mouse studies were reviewed and approved by the Institutional Animal Care and Use Committee (IACUC) at the Boston Children’s Hospital, and all mouse experiments were performed following the guidance of the Association for Research in Vision and Ophthalmology (ARVO) for the ethical use of animals in ophthalmic and vision research. Both male and female mice were used for experiments. C57BL/6J mice (Jackson Laboratory, stock # 000664) were used. The floxed *Socs3 (Socs3 f/f)* mouse line was generously provided by Dr. A. Yoshimura [31]. *Nestin-Cre* mice (Jackson Laboratory, stock # 003771) were crossed with *Socs3 f/f* mice to generate *Socs3 f/f; Nestin-Cre* mice.

### Laser-induced CNV model

Laser-induced CNV was generated using Micron IV Image-Guided Laser System (Phoenix) [32]. Mice were anesthetized using intraperitoneal ketamine/xylazine; pupils were dilated with 1% tropicamide. Four laser burns were placed in each eye. BoNT/A (BOTOX, Allergan, 0.125 unit per eye) or saline was administrated intravitreally to C57BL/6J mice immediately after laser exposure, designated day 1. 6 ~ 8-week-old mice were randomly divided into two groups: control and treatment. Mice were euthanized at the indicated days after laser exposure. Eyes were collected and fixed in 4% paraformaldehyde (Fisher Scientific, AAJ19943K2) in 0.01 M PBS for 1 h. The retinal pigment epithelium-sclera-choroid complex was dissected and permeabilized with 0.2% Triton X-100 in 50mM PBS for 1 h. Isolectin GS-IB4 (ThermoFisher Scientific, I21411, RRID: AB_23146) was used to stain the CNV lesions for phenotypical analysis or with indicated antibodies. After washing with PBS, the retinal pigment epithelium-sclera-choroid complex was whole-mounted onto slides using mounting medium (Vector Labs, H-1000-10). The images were taken using AxioObserver.Z1 microscope (Zeiss) or the Zeiss 700/710/980 confocal microscopes. Images for CNV lesions were quantified by masked researchers using ImageJ. Exclusion criteria for laser-induced CNV lesions were used as described [32].

### Fundus fluorescein angiography and quantification

Fundus fluorescein angiography (FFA) was performed as described [32]. In brief, mice were anesthetized and injected intraperitoneally with fluorescein AK-FLUOR (Akorn) at 5 µg/g body weight. Fluorescent fundus images with dilated pupils were taken with a retinal imaging microscope (Micron IV; Phoenix Research Laboratories) at 1, 3, 4, 5, 6, 7, 8, and 10 min after fluorescein injection. The FFA images were quantified using ImageJ.

### Immunohistochemistry

Immunostaining was performed as described [30]. Briefly, eyes were enucleated from the mice, fixed, and permeabilized. The whole-mounted retinas or cross sections were stained with antibodies and imaged using a confocal laser scanning microscope (Zeiss LSM980). Antibodies used in this study included Isolectin GS-IB4 (ThermoFisher Scientific, I21411, RRID: AB_23146), IBA1 (Wako, 019-19741, RRID: AB_839504), SOCS3 (Cell Signaling, 2923, RRID: AB_2255132), GFAP (Abcam, ab4674, RRID: AB_304558), and DAPI in an anti-fade mounting medium (Vector Labs, H-1200-10) for nuclear staining.

### Oxygen induced retinopathy (OIR) mouse model

OIR was induced in neonatal mice [33]. In brief, neonatal mice were placed with a nursing female mouse and exposed to 75% oxygen from postnatal day (P) 7 to P12 then returned to room air until P17. The exclusion criteria for the OIR mouse model were used as described [34]. Eyes were dissected at P13 and sectioned for immunostaining with SOCS3 (Cell Signaling, 2923, RRID: AB_2255132). Retinas were collected at P14 for gene expression.

### Intravitreal injection

Intravitreal injections were performed under a dissection microscope using a angled 35-gauge glass pipette controlled by a FemtoJet microinjector (Eppendorf) and inserted into the vitreous 1 mm posterior to the corneal limbus. Approximately 0.5 µl of solution containing 0.125 Unit of BoNT/A (BOTOX®) or saline was introduced into the vitreous. For the laser-induced CNV mouse model, intravitreal injections were performed following laser exposure. For the OIR mouse model, intravitreal injections were performed at P2. Mouse eyelids was opened after applying Betadine, followed by water and then 70% ethanol using cotton swabs. A blade was used to gently create an incision in the eyelid at the forming crease. After injection, curved forceps were employed to slowly close the eyelid, mice were then placed on a circulating water blanket to maintain warmth. The mice were then subjected to the OIR model.

### RNA isolation and quantitative real-time PCR

Total RNA was extracted from mouse choroid/retina using Quick-RNA™ Miniprep Kit (Zymo Research, R1054). cDNA was synthesized using iScript™ cDNA Synthesis Kit (Bio-Rad, 1,708,890). Quantitative real-time PCR was performed using SYBR Green PCR Master Mix (Apex Bio, K1070).

### Statistical analysis

GraphPad Prism was used for statistical analyses. Results are presented as mean ± Standard Error of the Mean (SEM). All experiments were repeated independently at least three times. Mann-Whitney test was used for two-group comparison. Kruskal-Wallis Dunn’s test was used for multiple-group comparison. *p* values < 0.05 were considered statistically significant.

## Results

### SNAREs proteins including SNAP25 and BoNT receptors were expressed in mouse retinas

**Fig. 1:**
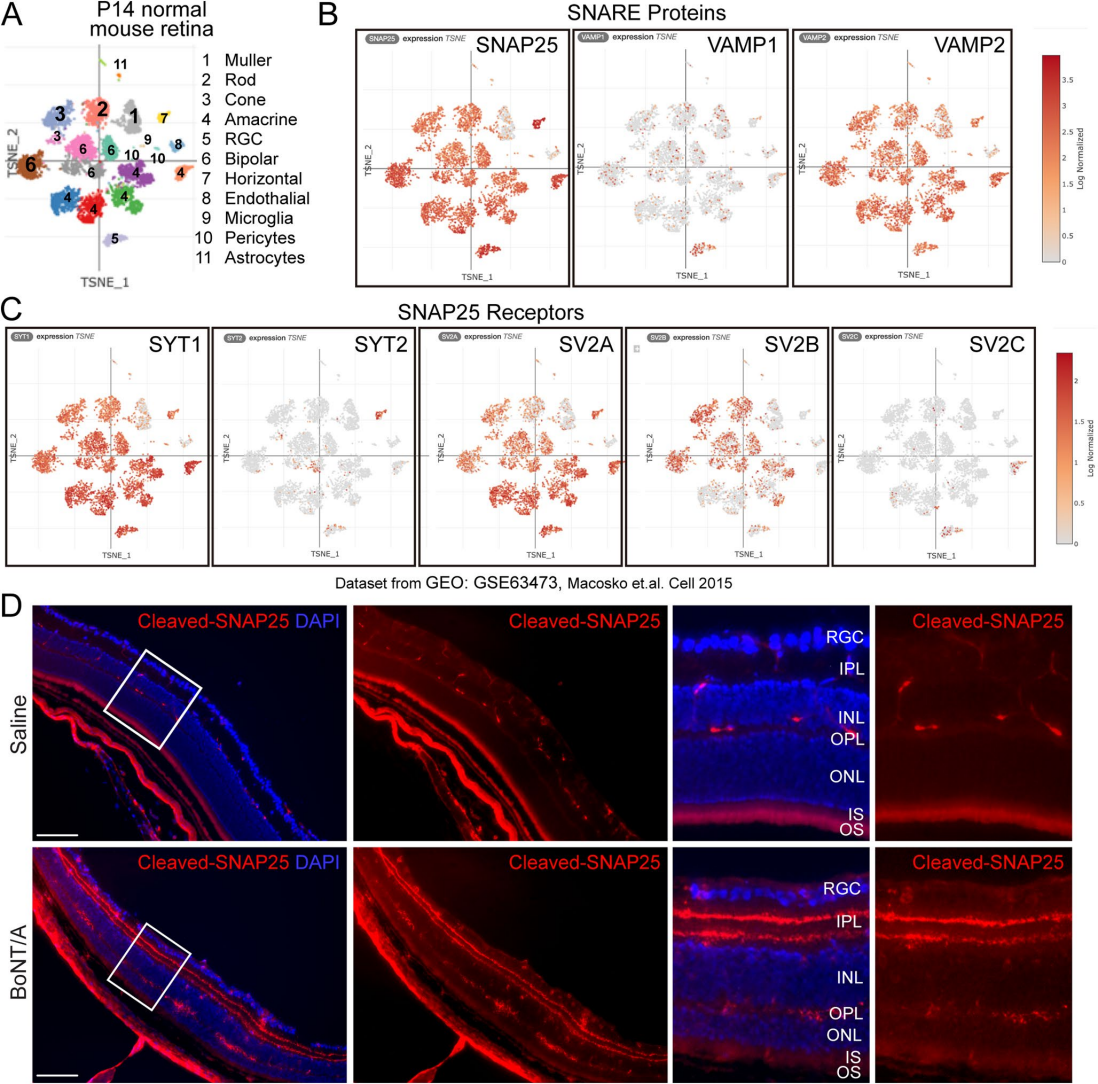
**The cleavage of SNAP25 protein by BoNT/A in mouse retina.** (**A**-**C**) scRNAseq dataset (https://singlecell.broadinstitute.org/single_cell/study/SCP301/c57b6-wild-type-p14-retina-by-drop-seq) showed the distribution of SNAREs proteins (SNAP25, VAMP1 and VAMP2) and the receptors of BoNTs (SV2A, SV2B, SV2C, SYT1, SYT2) in mouse retina. (**D**) BoNT/A or saline was intravitreally injected into mouse eyes of wild type mice and the retinas were dissected at 7 days after injection; cleaved-SNAP25 antibody (red) was used for immunostaining on retinal cross-sections. DAPI (blue) is for nuclear staining. RGC, retinal ganglion cells; IPL, inner plexiform layer; INL, inner nuclear layer; OPL, outer plexiform layer; ONL, outer nuclear layer; IS, inner segment; OS, outer segment. Scale bar: 100 µm

In a published scRNAseq dataset [35], tSNE plots showed the distribution of the known receptors of BoNTs (Syn, SV2A, SV2B, SV2C, Syt1, and Syt2) and SNARE proteins (SNAP25, VAMP1 and VAMP2) in mouse retina (Fig. 1, A-C). SNAP25, SYT1, SV2A, SV2B, and VAMP2 were broadly expressed in retinal neurons including rod and cone photoreceptors at P14. The distribution of SNARE proteins including SNAP25 and the receptors of BoNTs in mouse photoreceptors suggested that the acetylcholine pathway may contribute to photoreceptor function.

### BoNT/A cleaved SNAP25 in mouse retina

We then examined the enzymatic cleavage of BoNT in mouse retinas. BoNT/A or saline was injected into the posterior vitreous humor of wild type mouse eyes. Retinas were dissected 7 days post-injection. Immunostaining of cleaved-SNAP25 and DAPI on retinal cross-sections showed that cleaved SNAP25 was mainly localized between the retinal ganglion cells and the inner nuclear layer, corresponding to the inner plexiform layer in the BoNT/A-treated retinas, but not in the saline-treated controls (Fig. 1D). Previous studies have shown high levels of SNARE complex proteins, specifically SNAP25, in cholinergic amacrine cells [36], predominantly in the inner plexiform layer. Taken together, the data indicates that BoNT/A cleaves the known SNARE protein complex, SNAP25, in the mouse retina.

A previous study reported [37] a case of bullous retinal detachment occurred following BoNT/A intraocular injection. Remarkable, the retinal detachment spontaneously resolved, and vision returned to baseline within 2 days, suggesting that BoNT/A (Botox®) is not toxic to human intraocular tissue at an appropriate dosage. The potency of BoNT/A (Botox®) is expressed as Units, with 1 Unit corresponds to 1 LD50 (lethal dose for 50% of the test subjects) in the mouse bioassay [38]. In mice, a series dilution of BoNT/A1 has been tested via local intramuscular injection. A lower dose of 0.15 Units of BoNT/A showed functional paralytic effects [39]. Figure 1D showed that 0.125 Units of BoNT/A by intravitreal injection can sufficiently cleave SNAP25 in mouse retina. Additionally, we confirmed no toxicity of 0.125 Units of BoNT/A on mouse visual function by electroretinogram (supplementary Fig. 1). Therefore, the dosage of 0.125 Units was chosen for all the following experiments.

### BoNT/A decreased pathological laser-induced CNV in mouse model

**Fig. 2:**
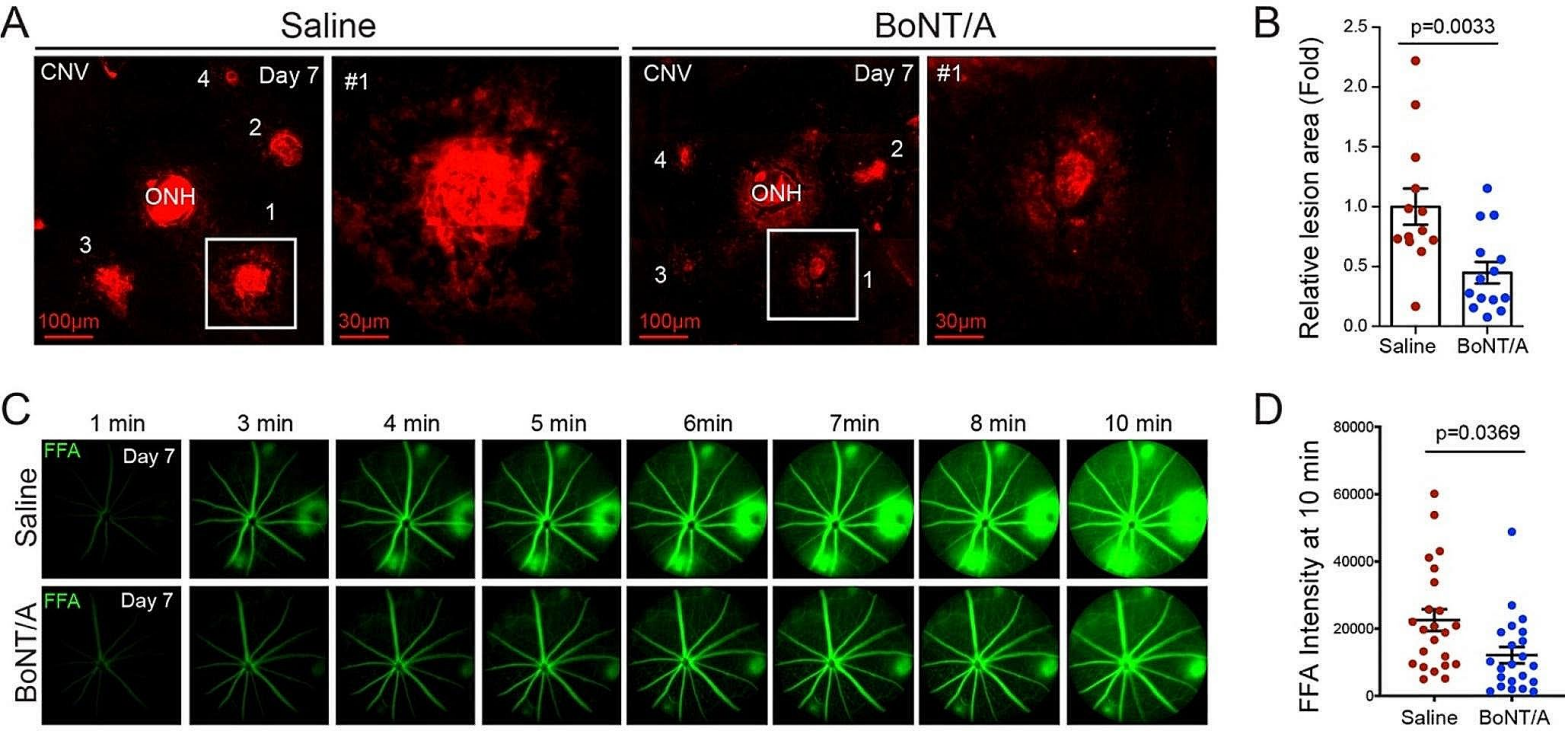
**BoNT/A suppressed laser-induced CNV and vessel leakage in mice.** (**A**) Representative Lectin-stained (red) choroidal flat mount images from BoNT/A- or saline- treated 6-8 weeks-old wild type mice with laser-induced CNV at day 7 and (**B**) quantification graph of CNV lesion areas (calculated as fold change vs saline control) (*n* = 13–15, *p* = 0.0033). (**C**) Representative FFA images at 1 min, 3 min, 4 min, 5 min, 6 min, 7 min, 8 min, and 10 min from BoNT/A- or saline-treated mice with laser-induced CNV at day 7. (**D**) The FFA intensity at 10 min was quantified using ImageJ (*n* = 21–23, *p* = 0.0369)

To examine the role of BoNT/A in pathological NV, we used a laser-induced CNV mouse model [32, 40, 41], commonly employed to mimic the angiogenic aspects of human NV AMD. This model allows for the study of CNV development mechanisms and the evaluation of potential treatments for NV AMD [42–46]. Adult mice underwent laser injury of the Bruch’s membrane, leading to the pathological growth of choroidal vessels into the subretinal space. BoNT/A was intravitreally injected into one eye, with saline as the control in the contralateral eye. Daily observations revealed no signs of inflammation such as redness, swelling, or other indicative ocular findings in either saline or BoNT/A injected eyes. CNV lesion areas in BoNT/A-treated mice showed ~ 32% decrease compared to saline controls (Fig. 2, A and B), indicating that BoNT/A limits pathologic NV progression in laser-induced CNV.

### BoNT/A reduced blood leakage in mice with laser-induced CNV in vivo

To further assess the impact of BoNT/A on pathological NV, leakage from laser-induced CNV vessels was examined. Eight-week-old C57BL/6J mice were subjected to laser-induced CNV, followed by intravitreal injection of BoNT/A or saline. At day 6 post-laser, vascular leakage was evaluated using FFA with a retinal imaging microscope (Micron IV, Phoenix). Fluorescein (Akorn) was intraperitoneally injected and FFA images were captured at 1-minute intervals for a total of 10 min. Representative FFA images and quantification graphs showed that BoNT/A significantly reduced leakage from CNV lesions compared to saline controls (Fig. 2, C and D). These results demonstrated that BoNT/A reduces vascular leakage in vivo in the laser-induced CNV mouse model.

### BoNT/A suppressed glial activation in the retina with laser-induced CNV

**Fig. 3:**
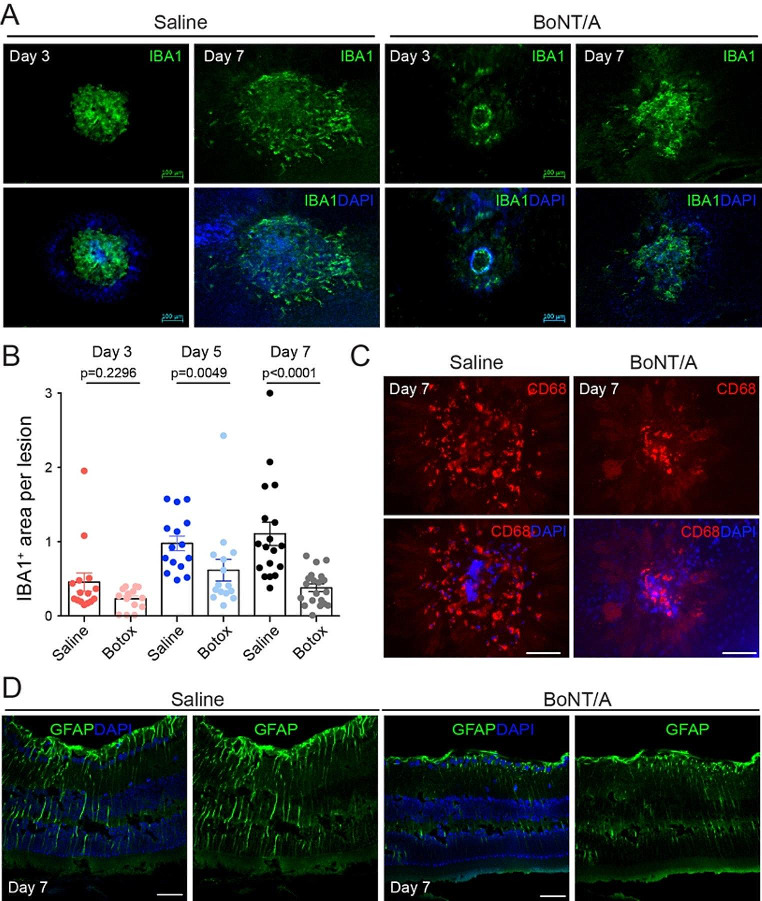
**BoNT/A suppressed glial activation during CNV.** (**A**) Representative choroidal flat mount images stained with IBA1 (green) and DAPI (blue) from BoNT/A- or saline-treated mice with laser-induced CNV at day 3 and day 7. Scale bar: 100 µm. (**B**) The areas of IBA1 staining in CNV areas at day 3, day 5, and day 7 post-laser were quantified (*n* = 15). (**C**) Representative choroidal flat mount images stained with CD68 and (**D**) retinal cross-section stained with GFAP and DAPI from BoNT/A- or saline-treated mice with laser-induced CNV at day 7 (*n* = 6). Scale bar: (C) 50 µm; (D) 100 µm

Microglia are the primary resident retinal immune cells associated with angiogenesis after an inflammatory insult [47, 48]. To assess the interaction between BoNT/A and glial cells under pathological conditions, 8-week-old C57BL/6J mice were subjected to laser-induced CNV, followed by intravitreal injection of BoNT/A into one eye and saline into the contralateral eye. Choroid/RPE/sclera complexes were stained for the microglia activation marker IBA1 at day 3, 5, and 7 post-laser and flat mounted, revealing decreased microglia recruitment to the site of laser-induced injury (Fig. 3, A and B). A significant decrease in number of IBA1-positive cells around CNV lesion areas was observed at day 5 and day 7 post-laser, but not at day 3 (Fig. 3B), indicating that the anti-angiogenic, therapeutic effects of BoNT/A may be time dependent. This time-dependent effect of BoNT/A is consistent with clinical observations that have found a 3 to 4 weeks lag to reach maximum therapeutic effect given its axoplasmic mechanism of movement [49]. Choroid/RPE/sclera complexes were further stained for the microglial/macrophage phagocytosis marker CD68, showing a decrease in microglial/macrophage phagocytosis (Fig. 3C). Additionally, cross-sections were stained for glial fibrillary acidic protein (GFAP) to visualize Müller glial/astrocyte activation, revealing decreased Müller cell and astrocyte activation in mice treated with BoNT/A (Fig. 3D). Taken together, these findings demonstrated that BoNT/A suppressed the activation of glial cells including Müller cell, astrocytes, and microglial cells in the retina with laser-induced CNV.

### BoNT/A induced SOCS3 expression during pathological NV

**Fig. 4:**
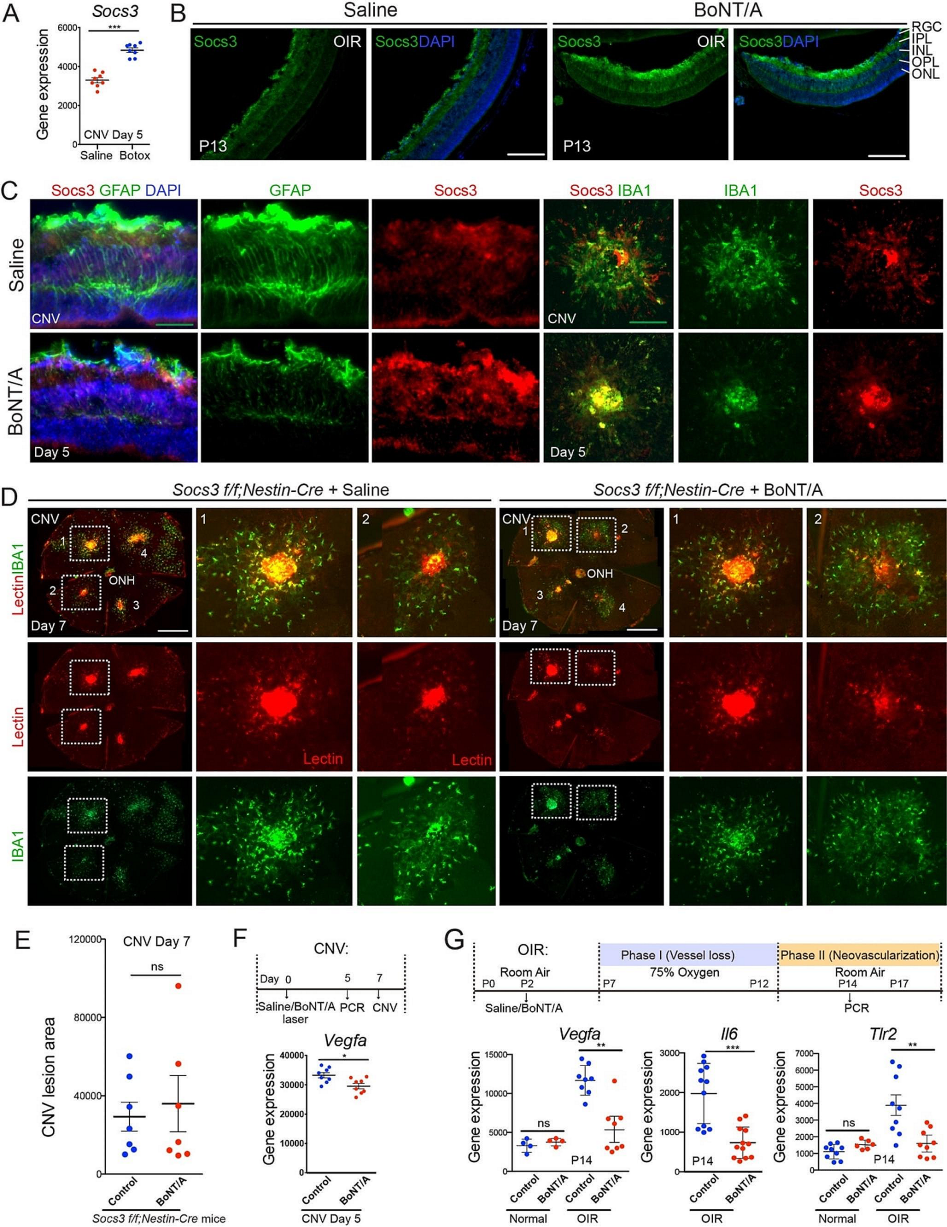
**BoNT/A induced SOCS3 expression during NV and neuronal/glial *Socs3 *deficiency abolished the protective role of BoNT/A in CNV.** (**A**) The mRNA expression of *Socs3* in choroid/retinas from mice with laser-induced CNV at day 5. The mice were intravitreally treated with BoNT/A or saline and subjected to laser-induced CNV at day 0 (*n* = 8). ***, *p* < 0.001. (**B**) Representative retinal cross-sections from BoNT/A- or saline-treated P13 OIR mice were stained with SOCS3 (green) and DAPI (blue) (*n* = 6). Scale bar: 200 µm. (**C**) Representative retinal cross-sections and choroidal flat mounts from BoNT/A- or saline-treated CNV mice at day 5 post-laser were stained with SOCS3 (red), GFAP (green), IBA1 (green), and DAPI (blue) (*n* = 6). Scale bar: 50 µm. (**D**-**E**) Representative choroidal flat mounts from BoNT/A- or saline-treated *Socs3 f/f; Nestin-Cre* mice with laser-induced CNV were stained with Lectin (red) and IBA1 (green) and quantification graph (*n* = 6). Scale bar: 500 µm. (**F**) The mRNA expression of *Vegfa* in choroid from mice with BoNT/A or saline treatment (*n* = 8). *, *p* < 0.05. (**G**) The mRNA expression of *Vegfa*, *Il6*, and *Tlr2* in P14 OIR retinas with BoNT/A or saline treatment (*n* = 4–8). **, *p* < 0.01; ***, *p*<0.001;  ns, no significance

We previously reported that SOCS3 regulates angiogenesis in both the laser-induced CNV model [30] and a ROP mouse model, oxygen-induced retinopathy (OIR) model [10]. To investigate if BoNT/A treatment influences SOCS3 expression during NV progression, we examined *Socs3* mRNA expression levels in the laser-induced CNV mouse model at day 5 post-laser. In BoNT/A-treated choroid/retinas tissues, *Socs3* mRNA expression was significantly induced compared to saline-treated controls (Fig. 4A), suggesting an upregulation of SOCS3 in response to BoNT/A treatment during NV. This finding was further validated in OIR mice. The immunostaining for SOCS3 on retinal cross-sections from BoNT/A- or saline-treated OIR mice at P13 showed a notable increase in SOCS3 expression in BoNT/A-treated OIR retina compared to saline controls (Fig. 4B). To future investigate SOCS3 induction in retinal glial cells by BoNT/A, we conducted co-staining of SOCS3 with GFAP for activated glial cells. Our findings reveal that SOCS3 was induced in both GFAP^+^ activated Müller glial cells and IBA^+^ activated microglia and macrophages (Fig. 4C). These results suggest that BoNT/A induces SOCS3 expression during pathological NV, implicating its potential regulatory role in BoNT/A-mediated anti-angiogenic effects.

### Angiogenic role of BoNT/A in CNV was attenuated in SOCS3 neuronal/glial deficient mice

We previously reported that neuronal/glial SOCS3 deficiency significantly increased pathological NV compared to littermate control mice [10]. To further elucidate the regulatory role of SOCS3 in BoNT/A-mediated anti-angiogenic effects, we generated neuronal and glial specific *Socs3* knockout mice (*Socs3 f/f; Nestin-Cre)* by breeding *Socs3* floxed mice (*Socs3 f/f)* with Nestin-Cre mice and treated these mice with BoNT/A or saline in a laser-induced CNV model. Choroid/RPE/sclera complexes from BoNT/A- or saline-treated *Socs3 f/f; Nestin-Cre* mice with laser-induced CNV were stained with Lectin for CNV lesion and IBA1 for activated microglia and macrophages. We observed that the IBA1-positive cells and CNV lesion size were comparable in BoNT/A- and saline-treated groups (Fig. 4, D and E), suggesting that neuronal/glial SOCS3 deficiency attenuated the inhibitory effect of BoNT/A on pathological CNV.

### BoNT/A suppressed expression of *Vegfa*, *Il6,* and *Tlr2* during NV

*Vegfa* mRNA in retinopathy models increases in Müller cells of the inner retina, contributing to pathological NV [9, 10, 50, 51]. To assess the effect of BoNT/A on *Vegfa* expression in mice with pathological NV, mice with CNV or OIR were treated with BoNT/A or saline. We observed decreased *Vegfa* mRNA expression in both CNV (Fig. 4F) and OIR (Fig. 4G) mice treated with BoNT/A compared to saline control treatment, indicating the potential inhibitory role of BoNT/A on *Vegfa* mRNA expression. Concurrently, *Socs3* expression was significantly increased and *Il6* mRNA expression was decreased in BoNT/A-treated mice compared to saline controls (Fig. 4, A-C, G), suggesting a relationship between BoNT/A treatment, increased *Socs3* expression, and decreased *Vegfa* and *Il6* mRNA expression. Moreover, BoNT/A is known to interact with TLR2 in microglia, exerting an anti-inflammatory effect [22, 23]. To investigate the involvement of TLR2 in the protective effects of BoNT/A in retinopathies, we assessed *Tlr2* mRNA expression in OIR retinas. We found that *Tlr2* retinal expression increased during OIR which was suppressed by BoNT/A treatment (Fig. 4G), implying potential involvement of TLR2 in the protective effects of BoNT/A in retinopathies. Further experiments are necessary to confirm this involvement and elucidate the underlying mechanisms.

## Discussion

BoNT/A is used therapeutically in many motor and autonomic disorders affecting both the central and peripheral nervous systems. Recent clinical case studies indicate that paraorbital injections with BoNT/A at conventional doses can improve neovascular leakage in AMD and diabetes [49]. Recent reports highlight the therapeutic potential of BoNT/A in pathological conditions involving glial cell activation [21, 52]. Therefore, exploring BoNT/A as novel therapeutic approach is crucial for addressing the current limitations of treatments for pathological NV. This study aimed to investigate BoNT/A as a potential anti-angiogenic agent and evaluate its effect on glial activation during retinopathy.

We used laser-induced CNV to investigate the potential of BoNT/A as an anti-angiogenic agent. Intravitreal injection of BoNT/A significantly reduced pathological CNV. Moreover, BoNT/A suppressed retinal glial activation during CNV. *Socs3* mRNA expression was increased in both CNV and OIR mice treated with BoNT/A, while *Vegfa* mRNA expression was suppressed in BoNT/A-treated CNV mice. Notably, the protective effect of BoNT/A was diminished in *Socs3* neuronal/glial deficient mice. This study suggests that a key mechanism underlying NV suppression by BoNT/A involves inhibition of glial cell activation. These findings support the hypothesis that BoNT/A induces SOCS3 expression in neuronal and glial cells, subsequently suppressing glial cell activation and thereby reducing VEGFA and other inflammatory factors.

Recent studies, such as Feng et al. [21], have demonstrated that the efficacy of a single intraplantar or intrathecal pre-administration of BoNT/A in rats subjected to partial sciatic nerve ligation, resulting in a reduction in pro-inflammatory cytokine induction in the spinal cord, dorsal horn, and dorsal root ganglia. To determine the direct effect of BoNT/A on microglia, Feng et al. performed in vitro experiments on lipopolysaccharide (LPS)-activated glial cells treated with BoNT/A. Their findings revealed that BoNT/A significantly inhibited the activation of LPS-treated microglia and reduced the release of pro-inflammatory cytokines, including TNFa, IL6, IL1, iNOS, and MIP-1, without affecting astrocyte activation. This latter result appears to contrast with our findings showing decreased Müller cell and astrocyte activation in mice exposed to BoNT/A, aligning with observation from other chronic pain models [53, 54].

Finocchiaro et al. demonstrated a marked reduction of spinal astrocyte activation in mice treated with BoNT/B in a study involving chronic constriction injury [55]. However, no significant difference was noted in the marker gene expression of resting and activated microglia were observed in chronic constriction injury mice treated with BoNT/B [55]. The discrepancies observed in the different BoNTs serotypes may depend on different targets of enzymatic activity for the toxins, namely, SNAP25 for BoNT/A and VAMP-2 for BoNT/B, and different expression of these targets in neuronal and non-neuronal cells.

Of note, cleaved-SNAP25 immunostaining was also observed in the outer plexiform layer and endothelial cell layer in the saline-injected eye. While little is known regarding SNAP25’s localization to endothelial cells, previous studies have identified synaptic docking proteins, like SNAP25, to be highly concentrated in the synapses of ribbon cell in the outer plexiform layer [56]. Despite this finding, Grabs et al. observed no immunoreactivity for SNAP25 in ribbon cell synapses, but heavy staining in the cell processes of the horizontal ribbon cell in the outer plexiform layer [57]. This differential staining suggests a variable staining pattern in these axons. While the immunostaining visible in the outer plexiform layer and endothelial cell layer most likely represents non-specific antibody staining, further investigation is necessary to confirm its differential staining pattern.

A unique property of BoNTs, as well as a limitation of its translation as a viable therapeutic option, involves its axoplasmic movement toward the central nervous system [58]. This property makes it capable of blocking activation and release of pro-inflammatory substances from glial cells via injection at distant sites but limits its functionality if the hypothesized retrograde transport of BoNTs cannot be controlled. It is worth further exploration to clarify the mechanism of action of BoNT/A, particularly a definitive clarification of the hypothesized retrograde transport of BoNT/A and whether the effects of peripheral BoNT/A on central spinal glial cells are solely due to direct interactions that occur after retrograde transport. Another clinically relevant question is how to best control retrograde transport as it identifies specific glial membrane receptors that allow BoNT/A to enter cells and the mechanism of action of BoNT/A inside glial cells.

The study delving into the therapeutic potential of BoNT/A in the context of AMD marks a significant stride in addressing current treatment limitations. AMD, particularly its NV form, causes rapid and severe vision loss, with current treatment approaches primarily centered on anti-VEGF therapies. However, concerns linger regarding the long-term outcomes. This study explores the efficacy of BoNT/A in mitigating CNV – a hallmark of NV AMD – through its interactions with retinal glial cells. Leveraging a mouse model of laser-induced CNV, the present study reveals a substantial reduction in pathological CNV following BoNT/A treatment. Notably, BoNT/A suppresses retinal glial activation, suggesting a potential mechanism for its therapeutic effects. The study also implicates the involvement of SOCS3 in mediating the actions of BoNT/A, further unraveling the intricate interplay between neuronal/glial-vascular communications in controlling CNV. These findings underscore BoNT/A as a promising avenue for AMD therapy, offering novel insights into its anti-angiogenic properties and paving the way for more targeted and effectve treatments for this prevalent cause of vision loss.

## Electronic supplementary material

Below is the link to the electronic supplementary material.

Supplementary Material 1

## Data Availability

No datasets were generated during the current study.
