# Supplementary material for: Botulinum neurotoxin serotype A inhibited ocular angiogenesis through modulating glial activation via SOCS3
Source: Angiogenesis. 2024 Jun 26;27(4):753–64. doi: 10.1007/s10456-024-09935-7 (PMC11564384; doi:10.1007/s10456-024-09935-7)
Supplement: Supplementary file 1 — Supplementary Material 1 [file 10456_2024_9935_MOESM1_ESM.docx]

**Supplemental Information**

**Methods**

**Electroretinogram**

Visual function was assessed by electroretinogram as described (1, 2)*.* These mice will be dark-adapted, anesthetized, pupils dilated, and corneas anesthetized. A Burian-Allen bipolar electrode (Hansen) will be placed on the cornea, and the ground electrode on a foot. ERG stimuli will be delivered using a Colordome Ganzfeld stimulator (Diagnosys). The saturating sensitivity of the photo-response will be estimated by fitting a model of the biochemical processes involved in the activation of phototransduction to the ERG a-waves (3). The saturating responses of b-waves were derived from the Naka–Rushton equation (4). All ERG data were presented as the log change from normal ($\Delta$LogNormal).

**References**

1. Sun Y*, et al.* (2017) Inflammatory signals from photoreceptor modulate pathological retinal angiogenesis via c-Fos. *The Journal of experimental medicine* 214(6):1753-1767.

2. Fu Z*, et al.* (2018) Photoreceptor glucose metabolism determines normal retinal vascular growth. *EMBO molecular medicine* 10(1):76-90.

3. Hood DC & Birch DG (1994) Rod phototransduction in retinitis pigmentosa: estimation and interpretation of parameters derived from the rod a-wave. *Investigative ophthalmology & visual science* 35(7):2948-2961.

4. Fulton AB & Rushton WA (1978) The human rod ERG: correlation with psychophysical responses in light and dark adaptation. *Vision research* 18(7):793-800.

**Figures**


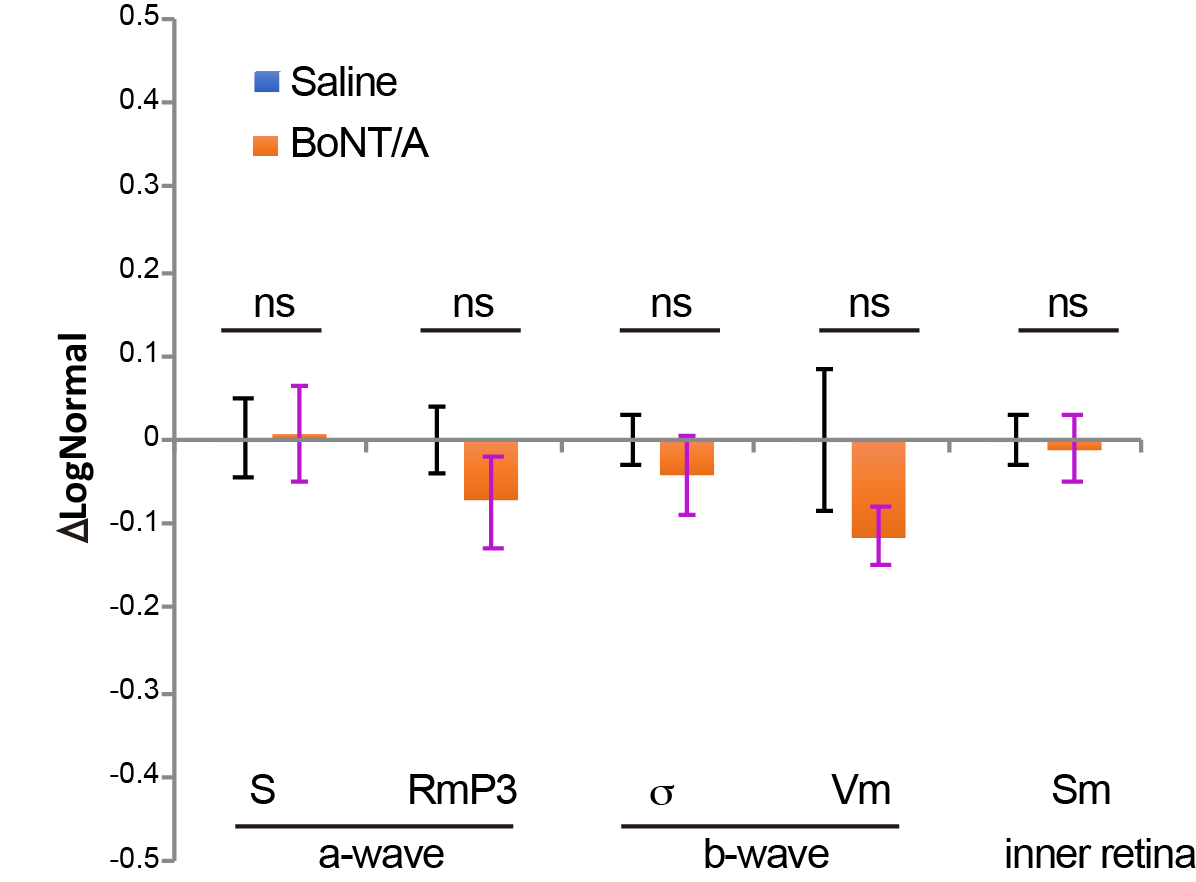


**Figure S1.** **Scotopic ERG for mice treated with BoNT/A**. The wild type mice were intravitreally injected with 0.125 Units of BoNT/A or saline and visual function was examined at 30 days post injection by ERG (n=15 eyes per group). ns, no significance.
